# Licochalcone B Ameliorates Liver Cancer via Targeting of Apoptotic Genes, DNA Repair Systems, and Cell Cycle Control

**DOI:** 10.22037/ijpr.2020.1101292

**Published:** 2020

**Authors:** Kadry Sadek, Tarek Abouzed, Sherif Nasr, Moustafa Shoukry

**Affiliations:** a *Department of Biochemistry, Faculty of Veterinary Medicine, Damanhur University, Egypt. *; b *Department of Biochemistry, Faculty of Veterinary Medicine, Kafr El-Sheikh University, Egypt. *; c *Department of Molecular Biology and Genetic Engineering, Faculty of Veterinary Medicine, Damanhur University, Egypt. *; d *Department of Physiology, Faculty of Veterinary Medicine, Kafr El-Sheikh University, Egypt.*

**Keywords:** Licochalcone B, Liver cancer, Signaling pathways, DNA repair system, Apoptosis

## Abstract

Apurinic/apyrimidinic endonuclease 1/redox factor-1 (APE1/Ref-1) is a ubiquitous multifunctional protein required in the DNA base excision repair pathway and a noteworthy reducing-oxidizing factor that regulates the activity of various transcription factors. Cyclin-dependent kinases (CDKs) assume a key role in directing the progression of the cell- cycle. The present study evaluated the synergistic efficacy of using licochalcone B (LCB) and fullerene C_60_ (FnC_60_) nanoparticles against diethylnitrosamine (DEN)-induced hepatocarcinoma in rats and relevant signaling pathways, with APE1/Ref-1 and CDK-4, as novel anti-cancer- targeting. LCB alone and in combination with FnC_60_ significantly decreased DNA fragmentation, oxidative DNA damage (8-hydroxy-2′-deoxyguanosine levels), APE1/Ref-1, CDK-4, retinoblastoma, B- cell lymphoma-2 (Bcl-2), B-cell lymphoma-xL (Bcl-xL), and β-arrestin-2 mRNA expression, and APE1/Ref-1 and CDK-4 protein expression. In contrast, these treatments significantly increased the expression of protein 53 (p53), Bcl-2-associated X protein (Bax), and caspase-3. These data suggest that LCB either alone or in combination with FnC_60_ elicited significant protective effects against DEN-induced hepatocarcinogenesis, which may have occurred because of the regulation of enzymes involved in DNA repair and cell-cycle control at S phase progression as well as the induction of apoptosis at the gene and protein expression levels. Furthermore, FnC_60_ potentiated the effect of LCB at the molecular level, possibly through targeting of cancerous cells.

## Introduction

Cancer is an important public health concern worldwide that has brought about an increasing number of deaths, and a huge number of patients are analyzed every year (1). Hepatocellular carcinoma (HCC) is one of the world’s most common cancers and causes almost one million deaths yearly (2). HCC is increasingly present all over the world because of the pandemic of obesity (3). The common treatment for HCC incorporates chemotherapy, radiation, and surgical resection; however, these treatments provide little promise for the restoration of well-being on account of poor results and severe side effects. Liver transplantation is thought to be the best option for patients with HCC. However, the low availability of organs limits this option for patients with HCC, and the high risk of tumor recurrence after transplantation further compromises its viability (4). Therefore, the development of more powerful and fewer side effects anti-tumor drugs, including non-synthetic agents, are needed to prevent or slow the process of hepatocarcinogenesis. Apurinic/apyrimidinic endonuclease 1 (APE1), the mammalian ortholog of *Escherichia coli *XthA (exonuclease III), is an essential protein that plays a crucial role in maintaining genome integrity. APE1 exhibits another vital capacity as redox factor-1 (Ref-1), which reductively activates transcription, factors including c-Jun, activator protein- 1, nuclear factor kappa B (NF-κB), the tumor- suppressor protein p53, hypoxia-inducible element 1α (HIF-1α), and paired box gene 8, which are involved in different cellular processes such as cell survival, cell signaling, and inflammatory (5). Past research has demonstrated the role of APE1/Ref-1 in the pathogenesis of numerous disorders, including liver fibrosis, liver cirrhosis, neuroinflammation, and different cancers (6-9). The proliferation of eukaryotic cells involves a methodical progression through four distinct phases of the cell cycle: G1, S, G2, and M (10). Cyclin-dependent kinases (CDKs) assume a key role in controlling cell-cycle progression and, to a large degree, oversee cell advancement from the developmental stages G1 and G2 into the stages associated with DNA replication (S) and mitosis (M) (11). Tumor initiation, promotion, and progression normally involve changes in programmed cell death. Several studies have demonstrated that dysregulation of programmed cell death is a critical causal variable in the development of HCC (12). Likewise, a growing body of evidence demonstrates that apoptosis induction is important in the chemoprevention of malignancy by dietary components (13). Flavonoids are among the most powerful and multifunctional substances in plants and have been shown to exhibit anti-cancer, anti-inflammatory, and hepatoprotective effects (14). Licochalcone B (LCB), which belongs to the retrochalcone family, is purified from Chinese licorice roots. Although the essential properties of LCB are still being investigated, studies have shown that LCB possesses powerful antioxidant and free radical scavenging properties as well as many other beneficial pharmacological activities, such as anti-inflammatory and cardioprotective effects (15, 16). Nanoparticle drug delivery systems have many interesting properties, including longer dissemination half-lives, enhanced pharmacokinetics, and decreased toxicity, especially at the systemic level (17). When combined with chemotherapeutic agents, nanoparticles can exploit the hyperpermeability and impaired blood flow of tumor vasculatures, thereby enhancing drug accumulation at the tumor site (18). Lately, researchers have investigated various ways to deliver multiple therapeutic agents using a single-medication nanocarrier because chemoresistance, the main cause of failed tumor treatment, may be prevented by using different medications. Tumor cells resist chemotherapy by overexpressing drug efflux pumps, expanding drug inactivation systems, increasing self-repairing capacity, or expressing altered drug targets (19). For some time, combination chemotherapy has been embraced in clinics as an essential cancer treatment regimen to reduce tumor drug resistance and improve treatment outcomes. Fullerene C_60_ (FnC_60_) is a nanoparticle that comprises 32 faces, including 12 pentagons and 20 hexagons. The carbon atoms are organized at 60 vertices of a truncated icosahedrons (20). The properties of FnC_60_ have been investigated extensively over the past decade, and the nanoparticle has been shown to have advantageous activity as a neuroprotectant as well as protective effects against cancer, inflammation, atherogenesis, and radiation (21). The present study aimed to evaluate the synergistic efficacy of using LCB in combination with FnC_60_ to prevent diethylnitrosamine (DEN)-induced HCC by assessing DNA fragmentation, oxidative DNA damage (8-hydroxy-2′-deoxyguanosine levels), mRNAs expression of different proapoptotic and anti-apoptotic markers, and mRNAs and protein expression of APE1/Ref-1 and CDK-4. The protective mechanism was further studied for related signaling pathways.

## Experimental

*Chemicals*

LCB (purity ≥ 98%) was purchased from Shanghai Li Chen Biotechnology Co., Ltd. (Shanghai, China). DEN and FnC_60_ were obtained from Sigma Chemical Co. (St. Louis, Missouri, USA). All other reagents were of analytical, high-performance liquid chromatography, or pharmaceutical grade.

*Preparation of FnC*_60_

FnC_60_ with a purity of more than 99.5% (MER Corporation, Tucson, Arizona, USA) was used. A total of 50 mg of FnC_60_Fn was dissolved in 10 mL of corn oil by mixing for two weeks at ambient temperatures, and the resulting blend was centrifuged at 5000 ×g for 30 min. The supernatant was sifted through a Millipore filter with a pore size of 0.25 mm (22).

*Animals and medicines*

Fifty male Wistar rats weighing 120–130 g were used in this study. The rats were treated following the Guide for the Care and Use of Laboratory Animals (National Institutes of Health. The rats were housed separately, and a standard laboratory diet and water were given ad libitum. The animal room was maintained at a constant temperature of 25 ºC with a relative humidity of 50% and a light/cycle of 12 h. After one week of the standard diet, animals were randomly assigned to five groups (n = 10). The rats in the DEN + LCB, DEN + FnC_60_, and DEN + LCB + FnC_60_ groups were pretreated with LCB and FnC_60_ (25 and 4 mg· (kg body weight)^-1^, either alone or in combination, by gastric tube for two weeks, whereas the animals in control and DEN groups received equivalent volumes of corn oil. Next, all the animals except for those in the control group received DEN (10 mg· (kg body weight)^-1 ^orally for 20 consecutive weeks and the rats in the DEN + LCB, DEN + FnC_60_, and DEN + LCB + FnC_60_ groups continued to receive LCB or FnC_60_ either alone or in combination until the end of the experiment. The dose of LCB was based on the results of a previous study by Teng *et* *al.* (23), while the dose of FnC_60_ was based on the results of Baati *et al.* (22) and the dosage of DEN was based on Zhang et al. study (24). The animals’ were weighed weekly, and the doses were adjusted as needed. Following 140 days of DEN treatment, the rats of all groups were anesthetized 24 h after the last treatment, and liver tissue was extracted and weighed. A portion of the liver was fixed in paraformaldehyde (10%) for histopathological examination, and the remainder was quickly frozen in liquid nitrogen prior to storage at -80 ºC.

*Determination of 8-OHdG in the liver*

DNA was separated from liver tissue using specialized kits. The levels of 8-OHdG were examined by ELISA. Briefly, an 8-OHdG antibody and sample DNA were added to an ELISA plate that had been pre-coated with 8-OHdG. The 8-OHdG in the sample competes with the 8-OHdG bound to the plate for the 8-OHdG antibody. The mean contents of 8-OHdG per microgram of DNA for each group were calculated for each sample. The percent DNA fragmentation in the liver tissue was measured using the colorimetrical diphenylamine assay.

*RNA purification and reverse transcription*

Total RNA was extracted from hepatic tissue using TRIzol reagent (Invitrogen Corp., USA) as per the manufacturer’s guidelines. The RNA was then dissolved in water containing diethyl pyrocarbonate. The concentration and purity of the RNA were determined using a Nanodrop spectrophotometer (Nanodrop 2000c, Thermo Scientific, USA). Complementary DNA was synthesized using a RevertAid First Strand cDNA Synthesis Kit as per the manufacturer’s protocol.

*Real-time PCR*

Gene expression levels in hepatic tissue were determined using real-time PCR. The primers were formulated by Sangon Biotech Co., Ltd. (Shanghai, China) (Table 1). All reactions were carried out using Maxima SYBR Green qPCR Master Mix in a MasterCyclerTM real plex 4 (Eppendorf, Westbury, New York, USA) with the following cycling conditions: initial denaturation at 92 ºC for 12 min, followed by 35 cycles of 10 s at 92 ºC, 30 s at 58 ºC, and 20 s at 70 ºC. The differences in gene expression among groups were calculated using the 2^-ΔΔ^^CT^ method, normalized to GAPDH and expressed as relative mRNA levels compared with the controls (25).

*Western blotting*

APE1/Ref-1 and CDK-4 protein expression were examined using western blot analysis. Protein extracts from liver tissue were prepared using a lysis buffer (50 mmol·L^- 1 ^Tris-HCl, pH 7.6, 0.5% Triton X-100, and 20% glycerol). The extracts were then subjected to centrifugation (15000 ×g, 15 min at 4 ºC). The supernatant fractions were assayed for protein concentration using Bradford reagent (Bio-Rad, Richmond, California, USA) and were then used for western blot analyses of APE1/Ref-1, CDK-4, and β-actin (Cell Signaling Technology, Beverly, Massachusetts A, USA). Horseradish peroxidase-conjugated IgG (Zymed, South San Francisco, California A, USA) was used as a secondary antibody.

*Liver histology*

For histopathology, paraffin-embedded sections approximately 5 µm thick were prepared, deparaffinized, rehydrated, stained with hematoxylin and eosin, and examined using an Olympus AX70 microscope (Olympus, Tokyo, Japan).

*Statistical analysis*

SPSS 13.0 was used for the statistical analysis. A chi-square test was performed to analyze the nodule incidence. Data were expressed as the mean ± SD and analyzed using a one-way ANOVA followed by Tukey’s test for multiple comparisons. Differences were considered significant at *P *< 0.05.

## Results

*Changes in body and liver weights*

**Figure 1:**
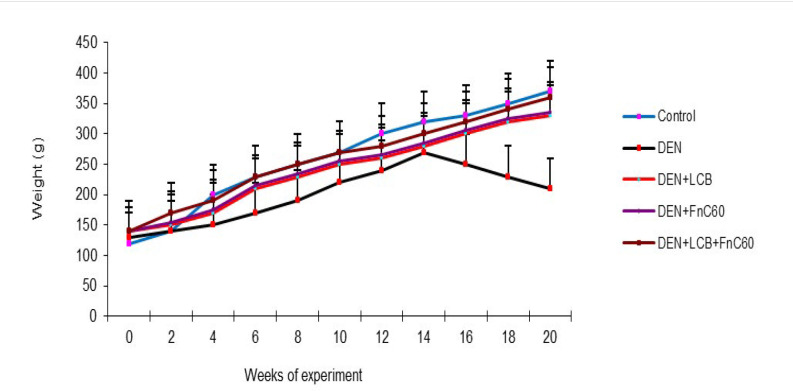
Changes in rat body weight. All values are expressed as mean **± **SD. Values with different letters significantly differed from the control group (*P* < 0.05)

^*^Number of rats in each group = 10.

**Figure 2:**
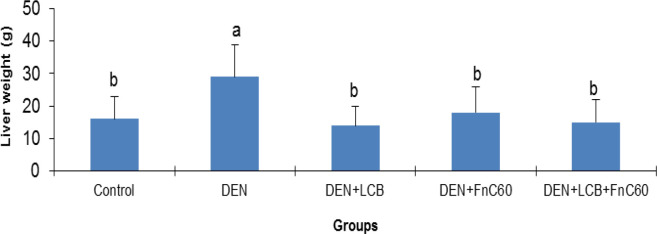
Changes in rat liver weight. All values are expressed as mean **± **SD. Values with different letters significantly differed from the control group (*P* < 0.05).

^*^Number of rats in each group = 10.

The effects of LCB and FnC_60_ on the bodyweight changes induced by DEN are summarized in Figure 1. After the first week of DEN administration, the rats began to exhibit slowed growth. In addition, with DEN treatment, the average weight gain gradually decreased. Following 98 days of DEN administration, the body weight in the DEN group began to decrease. After 140 days, the weights of the rats in the control group had increased to approximately three times their initial values, whereas the weights of the rats in the DEN group were only about 1.61- times their initial values. However, the LCB and FnC_60_ treatments significantly restored the body weight gain of the rats, and the DEN + LBCB + FnC_60_ group showed body weights closer to those of the control group. Figure 2 shows the liver weights of the different groups of animals. The liver weight in the DEN group was greater than that in the control group (*P *< 0.05). The administration of LBCB and FnC_60_ reduced the liver weight: in groups treated with LCB, FnC_60_, or their combination, the liver weight was lower than that of the DEN group (*P *< 0.05) and comparable to that of the control group.

*LCB and FnC*_60_ *inhibited DEN-induced formation of 8-OHdG and DNA fragmentation in the liver*

**Figure 3:**
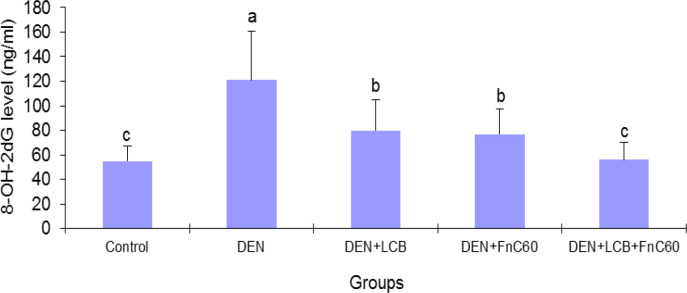
Effect of LCB and FnC_60_ on the 8-OHdG level in DEN-induced HCC. All values are expressed as mean **± **SD. Values with different letters significantly differed from the control group (*P* < 0.05).

^*^Number of rats in each group = 10.

**Figure 4:**
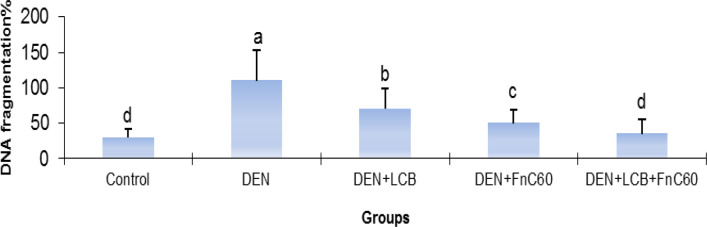
Effect of LCB and FnC_60_ on DNA fragmentation% in DEN-induced HCC. All values are expressed as mean **± **SD. Values with different letters significantly differed from the control group (*P* < 0.05)

^*^Number of rats in each group = 10.

As illustrated in Figure 3, after 140 days of DEN treatment, the 8-OHdG content increased by 2.31-fold (*P* < 0.01) in relation to the control group’s content. However, treatment with LCB and FnC_60_ attenuated the increase in 8-OHdG induced by DEN. The 8-OHdG content was significantly (*P *< 0.05) decreased by about 33.8%, 36.3%, and 53.7% in the DEN + LBCB, DEN + FnC_60_, and DEN + LBCB + FnC_60_ groups, respectively, compared with the DEN group, and the level in the DEN + LCB + FnC_60_ group was not significantly different from that in the control group. Also, the percent DNA fragmentation was significantly increased in the DEN-treated group compared to the control group, while it was significantly decreased in the DEN + LCB, DEN + FnC_60_, and DEN + LBCB + FnC_60_ groups compared with the DEN group and were not significantly different between the DEN + LCB + FnC_60_ group and the control animals (Figure 4).

*LCB and FnC*_60_ *augmented cell death in the livers of rats with DEN-induced HCC*

**Figure 5:**
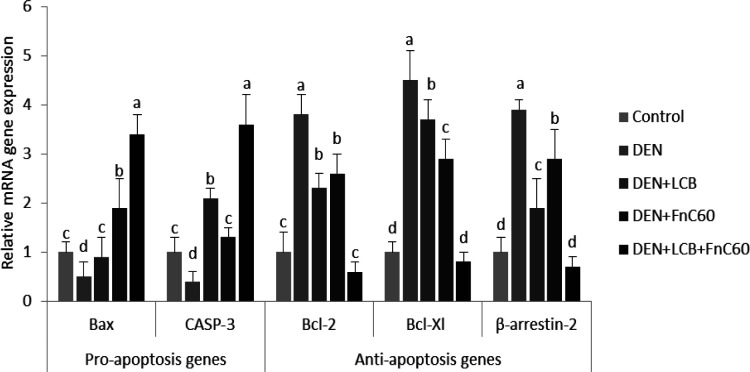
Effect of LCB, FnC_60_, and DEN on the mRNA level of Bcl-2, Bcl-xL, Bax, CASP-3, and β-arrestin. The mRNA levels were quantified with GAPDH as an internal control. All values are expressed as mean **± **SD. Values with different letters significantly differed from the control group (*P* < 0.05)

^*^Number of rats in each group = 10.

Because dysregulation of cell death is a crucial factor in the origination of hepatocarcinoma, we further quantified the gene expression of mediators of apoptosis (12). As illustrated in Figure 5, the real-time PCR data revealed upregulation of gene expression of the anti-apoptotic proteins B-cell lymphoma 2 (Bcl-2), B-cell lymphoma xL (Bcl-xL), and β-arrestin 2 in the DEN group compared with the control group (*P *< 0.05). However, significant decreases in the mRNA expression levels of Bcl-2-associated X protein (Bax) and caspase-3 (CASP-3) were observed in the DEN rats compared with the control animals. Therefore, the Bcl-2/Bax ratio was significantly increased in the DEN rats compared with the control group, Treatment with LCB and FnC_60_ obviously decreased the expression of Bcl-2 and Bcl-xL and augmented the expression of Bax and CASP-3 compared with levels in the DEN group. The Bcl-2/Bax ratios were significantly decreased in the LCB and FnC_60_ groups compared with the DEN group.

*LCB and FnC*_60_ *downregulated APE1/Ref-1 and upregulated p53 mRNA expression in the livers of DEN-treated rats*

**Figure 6:**
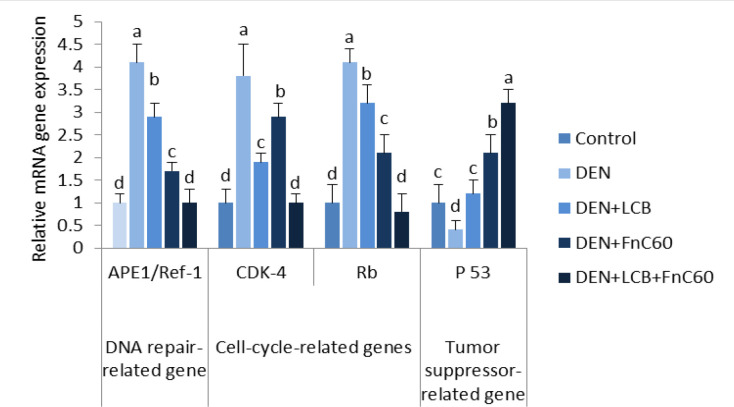
Effect of LCB, FnC_60,_ and DEN on the mRNA level APE-1/Ref-1, P53, Cdk-4, and Rb. The mRNA levels were quantified with GAPDH as an internal control. All values are expressed as mean **± **SD. Values with different letters significantly differed from the control group (*P* < 0.05)

^*^Number of rats in each group = 10.

As illustrated in Figure 6, the quantitative real-time PCR results revealed a significant increase in the mRNA expression levels of APE1/Ref-1 and a significant decrease in the mRNA expression level of p53 in the DEN group compared with the control group (*P *< 0.05). However, the administration of LCB and FnC_60_ markedly decreased the expression of APE1/Ref-1 mRNA and increased the expression of p53 mRNA compared with expression levels in the DEN group. The APE1/Ref-1 mRNA expression level in the DEN + LCB + FnC_60_ group was not significantly different from that of the control groups. 

*LCB and FnC*_60_ *downregulated CDK-4 and retinoblastoma (Rb) mRNA expression in the livers of DEN-treated rats*

As shown in Figure 6, the PCR results showed a significant increase in the mRNA expression levels of CDK-4 and Rb in the DEN group compared with the control group (*P *< 0.05). However, the administration of LCB and FnC_60_ markedly decreased the expression of CDK-4 and Rb mRNA compared with levels in the DEN group, and levels in the DEN + LCB + FnC_60_ group were not significantly different from those in the control group.

*LCB and FnC*_60_ *downregulated APE1/Ref-1 and CDK-4 protein expression in the livers of DEN-treated rats*

**Figure 7:**
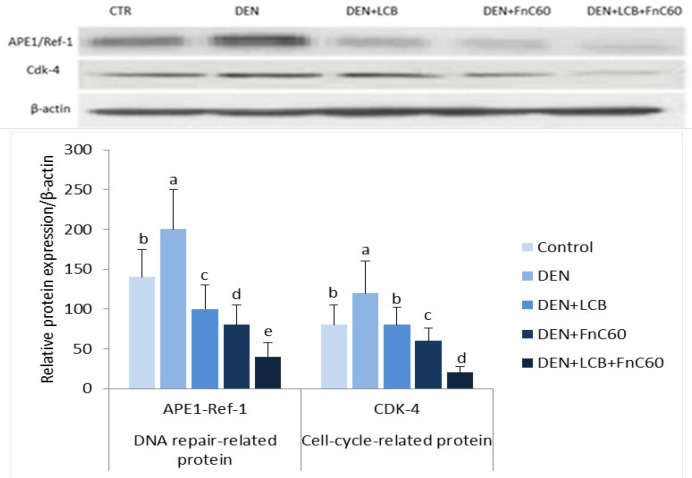
Effect of LCB, FnC_60, _and DEN on the protein expression level of APE-1/Ref-and Cdk-4 in liver tissue by Western blot analysis. The protein levels were quantified with β-actin as an internal control. All values are expressed as mean **± **SD. Values with different letters significantly differed from the control group (*P* < 0.05)

^*^Number of rats in each group = 10.

As shown in Figure 7, the western blot results revealed a significant increase in the protein expression levels of APE1/Ref-1 and CDK-4 in the DEN group compared with the control group (*P* < 0.05). However, the administration of LCB and FnC_60_ markedly decreased the expression of APE1/Ref-1 and CDK-4 compared with levels in the DEN group and the control group.

*Histological changes in the liver*

**Figure 8:**
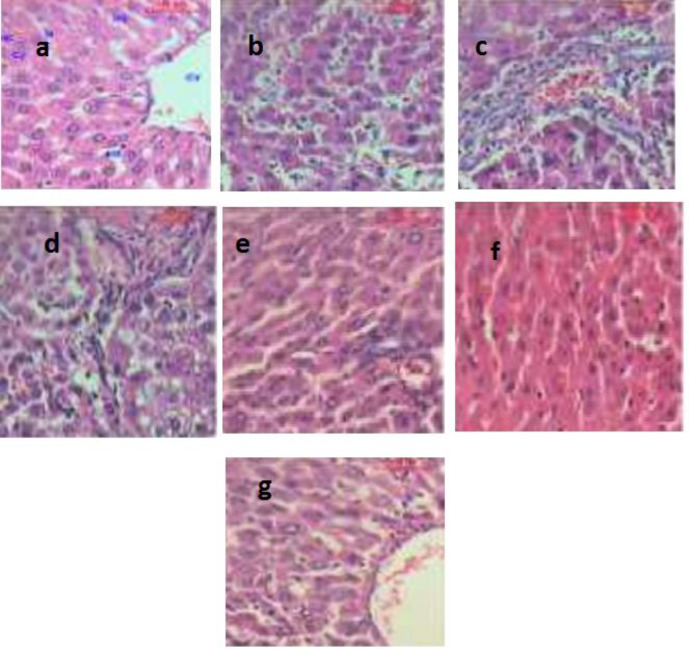
It shows photomicrographs (H&E X400) of various liver tissues from control and experimental animals. (a) shows the architecture of a hepatic lobule of control rats. The central vein (CV) lies at the center of the lobule surrounded by the hepatocytes (HC) with strongly eosinophilic granulated cytoplasm (CY) and distinct nuclei (N). Between the strands of hepatocytes, the hepatic sinusoids are shown (HS), (b) shows the area of the aberrant hepatocellular phenotype of DEN group with variation in nuclear size, hyperchromatism, and irregular sinusoids, (c) shows abnormal hepatocellular histology of DEN group with prominent hyperbasophilic preneoplastic focal lesions and eosinophilic clear cell foci, (d) shows trabeculae of hepatocellular carcinoma in DEN group consists of highly pleomorphic tumor cells and degenerated tumor cells, (e) shows the portal lobules of DEN + LCB group that appear more or less like the control one. Notice the activated Kuppfer'cells, (f) shows hepatic lobule of DEN + FnC_60_ group resembling control one, (g) shows many of the hepatocytes in DEN + LCB + FnC_60 _group appear more or less like normal

Representative histological images of the liver for all groups are shown in Figure 8. The hepatic tissue of the control group exhibited normal hepatocyte architectures (Figure 8a). In the rats treated with DEN, the liver sections showed areas of aberrant hepatocellular phenotypes that included nuclear size variations, hyperchromatism, and irregular sinusoids (Figure 8b). Also, abnormal hepatocellular histology with prominent hyperbasophilic preneoplastic focal lesions and the presence of eosinophilic and clear cell foci was also observed (Figure 8c). Finally, examining the livers of rats treated with DEN showed the trabeculae of HCC, including highly pleomorphic tumor cells and degenerated tumor cells. These cells were characterized by eosinophilic cytoplasm, large or binucleated nuclei, and prominent nucleoli. Intercellular bile canaliculi were also present (Figure 8d). These histopathological appearances were obviously lessened in the livers of rats treated with LCB and FnC_60_, which appeared similar to those of the control animals. Furthermore, histological analysis of the livers of rats treated with LCB and FnC_60_ revealed significant improvement in the hepatocellular architecture compared with that of the DEN group (Figures 8e-8g).

## Discussion

Lately, obvious considerations have been interested in using the antioxidants as possible targets for mitigating diverse maladies due to their specific natural actions and low toxicity (26-36).

In the present study, the combination of LCB and FnC_60_ reduced histopathological damage, inhibited oxidative DNA damage, and decreased DNA fragmentation induced by DEN. Furthermore, we found that LCB and FnC_60_ upregulated proapoptotic signals and downregulated anti-apoptotic signals. Interestingly, the tested compounds down-regulated the gene and protein expression of enzymes involved in DNA repair and cell-cycle progression. These results provide strong evidence that LCB and FnC_60_ act synergistically to prevent the development of DEN-induced HCC. Oxidative DNA damage might be a critical event in the development of malignancy (37). DEN, a potent hepatocarcinogen in rats, may exert its effects by altering DNA structure, particularly by generating alkyl DNA adducts and producing chromosomal abnormalities and micronuclei in the liver (38). To further evaluate the role of LCB and FnC_60_ in the inhibition of DEN-induced HCC, we determined the contents of 8-OHdG, a DNA lesion induced by reactive oxygen species (ROS) and an important indicator of oxidative DNA injury caused by advanced age, tumors, and chronic diseases (39). The results clearly indicated that treatment with LCB and FnC_60_ significantly reduced hepatic 8-OHdG content and percent DNA fragmentation in relation to the levels observed in DEN-treated rats, which suggests that LCB and FnC_60_ prevented DEN-induced DNA damage. Tumor initiation, promotion, and progression usually involve changes in programmed cell death. Studies have shown that dysregulation of cell death is imperative for HCC advancement and that induction of apoptosis is important in the chemoprevention of malignancy by dietary components (12, 13). Thus, we evaluated changes in the mRNA expression level of mediators of apoptosis. The Bcl-2 protein family regulates proapoptotic factors and has a vital role in determining the capacity of cells to experience cell death (40). Bax is normally found in the cytoplasm but translocates to the mitochondria to promote cell death; however, the activity of Bax is neutralized by anti-apoptotic factors such as Bcl-2 (41). Numerous tumor cells avoid apoptosis by upregulating Bcl-2 and Bcl-xL expression (42). In the current work, LCB and FnC_60_ upregulated Bax expression and downregulated Bcl-2 and Bcl-xL expression, in contrast with the changes observed in rats treated only with DEN. The ratio of Bcl-2 to Bax, rather than the levels of the individual proteins, is thought to be a basic determinant of cell survival or demise (43). A decrease in the Bcl2/Bax ratio will initiate cell death (44). In the current study, the ratio of Bcl-2 to Bax was substantially reduced by treatment with LCB and FnC_60_, which may have triggered apoptosis amid the hepatocarcinogenesis induced by DEN. A number of studies have demonstrated that an important mechanism by which capsaicin, zerumbone, matrine, and leptin prevent HCC involves reducing Bcl-2/Bax ratios (45). CASP-3 activation is crucial for the execution of apoptosis and is a specific event in both the internal and external apoptotic pathways (46). In the current experiment, DEN exposure reduced CASP-3 expression, while LCB and FnC_60_ increased CASP-3 expression. Cell death induced by PTX-2 in human HCC is associated with downregulation of Bcl-2 and Bcl-xL, upregulation of Bax, and activation of CASP-3 (47). Thus, LCB and FnC_60_ may have triggered apoptosis through downregulation of Bcl-2 and Bcl-xL and upregulation of Bax and CASP-3, and these components might be essential for the prevention of HCC. The protein β-arrestin-2 was initially recognized as a terminator of G protein-coupled receptor signaling and has been shown to have novel capacities as a signal transducer in different pathways (48). With these activities, β-arrestin 2 may prevent apoptotic signaling by activating the anti-apoptotic pathway (49). In the current study, β –arrestin 2 transcription was increased in DEN-treated rodents and this may have had an anti-apoptotic effect related to upregulation of Bcl-2 and Bcl-xL and downregulation of Bax and CASP-3. Conversely, the LCB and FnC_60_ treatments reduced β-arrestin 2 mRNA expression levels, thereby promoting apoptotic signaling through upregulation of Bax and CASP-3. Moreover, β -arrestin-2 has a role in the Wnt signaling pathway, which is frequently imbalanced in HCC (50). Increased ROS generation is a mechanism by which certain exogenous chemicals instigate apoptosis; therefore, antioxidants can suppress apoptosis (51). In addition, certain chemicals prevent tumors by increasing antioxidant activity and causing cell death. For instance, polyphenols, lycopene, and moringa *oleifera *have noteworthy chemo-prophylactic effects, counteracting disease through their antioxidant activities and induction of cell death (51). Therefore, it is plausible that LCB and FnC_60_ have powerful antioxidant capacities as well as proapoptotic effects. Pretreatment with LCB and FnC_60_ reduced ROS generation, which was reflected in decreased 8-OHdG levels, without hindering the proapoptotic activities of LCB and FnC_60_. This fact indicates that the mechanisms of HCC prevention by LCB and FnC_60_ has multiple components. The control of CDK action happens at numerous levels, including the addition and removal of phosphate from CDK, cyclin formation and destruction, the transcription, degradation, and accessibility of protein inhibitors, and the subcellular localization of these different components (10). Progression through the G1-/S transition requires the addition of phosphate to Rb by CDK-4 or the highly homologous kinase CDK-6 complexed with its activating subunits, the D type cyclins (D1, D2, and D3) (52, 53). Major phosphorylation of Rb lessens its capacity to quell gene expression through the E2F family of transcription factors and therefore permits the synthesis of many genes, the protein products of which are important for DNA division (54). Accordingly, the activity of CDK-4 or CDK-6 manages a basic checkpoint for the G1-/S transition and cell division (55). More than 90% of human cancers have biochemical and genetic adaptations that allow them to circumvent the mechanisms that control this checkpoint (56). The irregularities include upregulation of CDK-4 enhancement of D-type cyclins; downregulation of p^16INK4A ^, an inhibitor of CDK-4; changes in CDK-4 that inhibit the action of p^16INK4A^ on the enzyme; and degradation or transformation of Rb (57). These distortions can trigger the loss of proliferative controls either by removing the checkpoint completely or by promoting inappropriate or increased CDK-4 activity, which brings about Rb hyperphosphorylation. The recurrence of these changes suggests that annulment of the G1 checkpoint and speeding up of the CDK-4/cyclin D pathway creates favorable circumstances for the multiplication and survival of cancer cells. In view of this, cyclin D–dependent kinases have long been considered prime targets for chemotherapy (58). Preliminary evidence suggests that inhibition of cyclin D–dependent kinase activity may prevent cancer development or at least reverse the altered phenotype. For instance, the reduction of cyclin D expression through missense mutation causes an accompanying decrease in cyclin D–dependent kinase activity and subsequently inhibits cancer development, nullifies tumorigenicity, or causes tumor cell death in specific cases (59). Other studies have demonstrated that upregulation of p16INK4A expression in tumor cells can inhibit proliferation and reduce tumorigenicity (60). These observations bolster the case for using CDK-4/6 as a target for tumor treatment and suggest that a specific inhibitor of these enzymes would produce a meaningful therapeutic response. Our results showed that DEN induces HCC through upregulation of both CDK-4 and Rb, and the administration of LCB and FnC_60_ downregulates CDK-4 and Rb. These novel discoveries provide new information on the anti-cancer potential of the tested compounds.

APE1/Ref-1 has been associated with several different human ailments, and its dysregulation, post-translational modification, or sequence polymorphisms can influence susceptibility toward these maladies, as observed for several types of cancer (9). In this study, DEN initiated treatment upregulated APE1/Ref-1 expression. Overexpression and increased enzymatic activities of APE1/Ref-1 have been associated with survival and chemoresistance in cancer cells (61). Phytochemicals may alter the repair and redox activities of APE1/Ref-1 (62). Resveratrol (trans-3′,4′,5-trihydroxystilbene), an essential polyphenolic anti-cancer agent present in grapes and other food sources, has shown antiproliferative activity against cancer cells *in-vitro *(63). Resveratrol also has a vital role in counteracting stress by regulating APE/Ref-1 (8). The antioxidant effect of LCB (23) and FnC_60_ (64) may be the mechanism by which these compounds reduce the expression of APE-1/Ref-1, which is principally activated by oxidative stress (8). APE1/Ref-1 also activates HIF-1α and p53 using its redox activity. Activation of the tumor-suppressor protein p53 by APE1/Ref-1 adds to the oxidative stress response. Activated p53 translocates to the nucleus to promote the transcription of p53-responsive genes, such as p21, cyclin G, and Bax genes (65). To elucidate the mechanisms underlying the protective effect of LCB and FnC_60_, we measured the protein expression of APE-1/Ref-1 and CDK-4. Western blot analysis showed that DEN treatment enhanced APE-1/Ref-1 and CDK-4 protein expression. However, LCB either alone or in combination with FnC_60_ decreased APE1/Ref-1 and CDK-4 protein expression, indicating that regulation occurs at both transcriptional and translational levels.

Changes at the molecular level were confirmed by histopathology. The histopathological images of livers of rats in the DEN group demonstrated a distorted hepatocellular phenotype that included nuclear size variations, hyperchromatism, and sporadic sinusoids with conspicuous hyperbasophilic preneoplastic central lesions and eosinophilic clear cell foci. Additionally, profoundly pleomorphic and degenerated tumor cells were observed. This finding was consistent with the results obtained by Sreepriya and Bali, who reported that the presence of atypical nuclei is a marker of HCC (66). Nevertheless, animals receiving LCB alone or in combination with FnC_60_ showed entryway regions and hepatic lobules comparable to those of the control animals, which demonstrated liver recovery. These results appear to show that FnC_60_ had a synergistic effect increasing the penetration and consequently the bioavailability of LCB. Biological membranes play critical roles in the homeostasis of all organisms because they segregate important activities between and within cells and tissues. Small hydrophobic molecules can partition across biological membranes down a concentration gradient, but hydrophilic molecules generally require some sort of selective transport system to cross the lipid bilayer. Previous studies have shown that certain compounds can facilitate the transport of polar molecules across biological membranes (67).

**Table 1:** Nucleotide sequences of the primers used in RT-PCR

**Gene symbol**	**GenBank** **accession no.**	**Primer (5'→3')**
Bcl-2	NM_016993.1	F: CCCCAGAAGAAACTGAACC R: GCATCTCCTTGTCTACGC
Bcl-XL	XM_017591479.1	F: CGTGGAAAGCGTAGACAAGG R: CAACAACCATGCCAGGAGAC
β-arrestin-2	NM_012911.1	F: CCACGTCACCAACAATTCTG R: TTGGTGTCTTCGTGCTTGAG
Bax	NM_017059.2	F: GTTGCCCTCTTCTACTTTGC R: ATGGTCACTGTCTGCCATG
Caspase-3	NM_012922.2	F: CTGGACTGCGGTATTGAGAC R: CCGGGTGCGGTAGAGTAAGC
APE-1/Ref-1	NM_024148.1	F: TGCTGTGTGGGGATCTCAAT R: CCAACATTCTTAGAGCGGGC
P53	NM_030989.3	F: CTCCTCTCCCCAGCAAAAGA R: GTAGACTGGCCCTTCTTGGT
Cdk-4	NM_053593.2	F: TGATGCGCCAGTTTCTAAGC R: CCACATATCCACAGGCGTTG
Rb	NM_017045.1	F: TCACATTCCTCGAAGCCCTT R: TGGAACTTCTCGGATGTCCC
GAPDH^*^	NM_017008.4	F: TCAAGAAGGTGGTGAAGCAG R: AGGTGGAAGAATGGGAGTTG

^*^Housekeeping gene.

## Conclusion

To our knowledge, the findings presented herein are the first to demonstrate the *in-vivo* synergistic effects of LCB and FnC_60_ against DEN-induced HCC. The chemo-prophylactic effect of LCB and FnC_60_ may be accompanied by regulation of the DNA repair system and the cell- cycle at S-phase progression and the induction of apoptosis at the gene and protein expression levels. Effective treatments for HCC are scarce; however, our findings suggest the potential utility of LCB and FnC_60_ in chemoprevention of HCC with APE1/Ref-1 and CDK-4 as therapeutic targets.
